# Ticlopidine in Its Prodrug Form Is a Selective Inhibitor of Human NTPDase1

**DOI:** 10.1155/2014/547480

**Published:** 2014-08-11

**Authors:** Joanna Lecka, Michel Fausther, Beat Künzli, Jean Sévigny

**Affiliations:** ^1^Département de Microbiologie-Infectiologie et d'Immunologie, Faculté de Médecine, Université Laval, Québec, QC, Canada G1V 0A6; ^2^Centre de Recherche du CHU de Québec, 2705 Boulevard Laurier, Local T1-49, Québec, QC, Canada G1V 4G2; ^3^Department of Surgery, Klinikum Rechts der Isar, Technische Universität München, 81675 Munich, Germany

## Abstract

Nucleoside triphosphate diphosphohydrolase-1 (NTPDase1), like other ectonucleotidases, controls extracellular nucleotide levels and consequently their (patho)physiological responses such as in thrombosis, inflammation, and cancer. Selective NTPDase1 inhibitors would therefore be very useful. We previously observed that ticlopidine in its prodrug form, which does not affect P2 receptor activity, inhibited the recombinant form of human NTPDase1 (*K*_*i*_ = 14 *μ*M). Here we tested whether ticlopidine can be used as a selective inhibitor of NTPDase1. We confirmed that ticlopidine inhibits NTPDase1 in different forms and in different assays. The ADPase activity of intact HUVEC as well as of COS-7 cells transfected with human NTPDase1 was strongly inhibited by 100 *µ*M ticlopidine, 99 and 86%, respectively. Ticlopidine (100 *µ*M) completely inhibited the ATPase activity of NTPDase1 *in situ* as shown by enzyme histochemistry with human liver and pancreas sections. Ticlopidine also inhibited the activity of rat and mouse NTPDase1 and of potato apyrase. At 100 *µ*M ticlopidine did not affect the activity of human NTPDase2, NTPDase3, and NTPDase8, nor of NPP1 and NPP3. Weak inhibition (10–20%) of NTPDase3 and -8 was observed at 1 mM ticlopidine. These results show that ticlopidine is a specific inhibitor of NTPDase1 that can be used in enzymatic and histochemistry assays.

## 1. Introduction

Extracellular nucleotides are released during different processes including exocytosis (e.g., platelets), shear stress (e.g., red blood cells), cell activation (e.g., platelets, endothelial cells), and cell lysis [1, 2]. Once released the effect of nucleotides is exerted via the activation of several specific receptors, namely, P2X1-7 and P2Y_1,2,4,6,11–14_, and perhaps also via cysLT1R, cysLT2R, and/or GPR17 [3, 4].

The action of nucleotides (ATP, ADP, UTP, and UDP) on P2 receptors is regulated by ectonucleotidases [5, 6]. Nucleoside triphosphate diphosphohydrolase-1 (NTPDase1) is the main ectonucleotidase at the surfaces of vascular endothelial cells, blood cells, and smooth muscle cells [7, 8]. NTPDase1 is present all along the cell surface and it was also observed in caveolae, a specialized structure of the plasma membrane [9, 10]. Similar to other ectonucleotidases, NTPDase1 catabolizes extracellular nucleotides [11].

By controlling extracellular nucleotides' levels, NTPDase1 affects various biological processes such as haemostasis [12, 13], vascular smooth muscle cell contraction [14, 15], pain perception [3, 16], angiogenesis, vascular permeability [17, 18], airway epithelial transport [19], endocrine secretion [20], neurotransmission and neuromodulation [21], inflammation, and immune reactions [11, 22–25]. An imbalanced ATP/ADP hydrolysis ratio was observed in patients with coronary artery disease and abdominal aortic aneurysm [26, 27] where NTPDase1 would be expected to be involved. The product of NTPDase1 activity, AMP, can be further catabolized by ecto-5′-nucleotidase to adenosine, the agonist of P1 receptors [28]. Adenosine is also involved in various functions regulated by ATP and most often exerts an opposite effect to ATP such as in the regulation of the vascular tone, cell migration, proliferation, and differentiation [29]. NTPDase1 inhibitors may therefore represent a valuable tool to potentiate various physiological actions of nucleotides and could also serve as potential drug candidates for the treatment of some diseases associated with functions of NTPDase1 such as in cardiovascular [11, 22, 27] and immune diseases [23, 30] and cancer [31, 32].

We previously observed that, by blocking endothelial cell NTPDase1 activity, the thienopyridines ticlopidine (Tyklid) and clopidogrel (Plavix) impaired platelet aggregation [33]. While clopidogrel is solubilized poorly in polar solvents, ticlopidine is easier to solubilize, making it a more convenient candidate for inhibition assays. It is noteworthy that ticlopidine (Tyklid) and clopidogrel (Plavix) are widely prescribed after heart attacks. As prodrugs they must be metabolically activated to the forms that irreversibly block platelet P2Y_12_ receptors [34, 35]. Although ticlopidine cannot obviously be used in a long-term basis as a therapeutic agent to block NTPDase1 in human (due to its catabolism by the liver to a P2Y_12_ antagonist) it can have several other advantages such as studying NTPDase1 functions. As several NTPDases have distinct functions, specific NTPDase inhibitors would be greatly valuable. For example, while NTPDase1 abrogates platelet aggregation and their recruitment in intact vessels via the hydrolysis of ADP, NTPDase2, by the hydrolysis of ATP to ADP, has the ability to facilitate platelet activation at sites of extravasation [7]. Indeed, while NTPDase1 is expressed by vascular endothelial cells, in touch with blood components, NTPDase2 is expressed in the subendothelium of veins and in the adventitial cells of arteries [36] which are exposed to platelets only after blood vessel breakage. In this study, we demonstrate that ticlopidine in its prodrug form can be used as a selective NTPDase1 inhibitor.

## 2. Materials and Methods

### 2.1. Materials

Aprotinin, nucleotides, apyrase grade VII, phenylmethanesulfonyl fluoride (PMSF), ticlopidine, and malachite green were purchased from Sigma-Aldrich (Oakville, ON, Canada). Tris was obtained from VWR (Montreal, QC, Canada). DMEM was obtained from Invitrogen (Burlington, ON, Canada). Fetal bovine serum (FBS) and antibiotics-antimycotics solution were from Wisent (St-Bruno, QC, Canada). Formalin and acetone were obtained from Fisher Scientific (Ottawa, ON, Canada). OCT freezing medium was purchased from Tissue-Tek, Sakura Finetk (Torrance, CA).

### 2.2. Plasmids

The plasmids used in this study have all been described in published reports: human NTPDase1 (GenBank accession number U87967) [37], human NTPDase2 (NM_203468) [38], human NTPDase3 (AF034840) [39], human NTPDase8 (AY430414) [40], mouse NTPDase1 (NM_009848) [12], rat NTPDase1 (NM_022587) [41], human NPP1 (NM_006208) [42], and human NPP3 (NM_005021) [43].

### 2.3. Cell Transfection and Protein Preparation

COS-7 cells were transfected with an expression vector (pcDNA3) containing the cDNA encoding for each ectonucleotidase using Lipofectamine (Invritrogen) and harvested 72 h later, as previously described [33]. For the preparation of protein extracts, transfected cells were washed three times with Tris-saline buffer at 4°C, collected by scraping in harvesting buffer (95 mM NaCl, 0.1 mM PMSF, and 45 mM Tris, pH 7.5), and washed twice by centrifugation (300 g, 10 min, 4°C). The cells were then resuspended in the harvesting buffer supplemented with 10 *μ*g/mL aprotinin to block proteinases and sonicated. Nucleus and large cellular debris were discarded by centrifugation (300 ×g, 10 min, 4°C) and the supernatant (thereafter called lysate) was aliquoted and stored at −80°C until used. Protein concentration in the lysates was estimated by Bradford microplate assay using bovine serum albumin as a standard [44].

### 2.4. Enzymatic Activity Assays

#### 2.4.1. NTPDases and Apyrase (EC 3.6.1.5.)

Activity was measured as described previously [5] in 0.2 mL of incubation medium (5 mM CaCl_2_ and 80 mM Tris, pH 7.4) or Tris-Ringer buffer (in mM, 120 NaCl, 5 KCl, 2.5 CaCl_2_, 1.2 MgSO_4_, 25 NaHCO_3_, 5 mM glucose, and 80 Tris, pH 7.4) at 37°C with or without ticlopidine. Ectonucleotidase lysates were added to the incubation mixture and preincubated at 37°C for 3 min. The reaction was initiated by the addition of 100 *μ*M ATP or ADP for NTPDases, with or without ticlopidine (100 *μ*M or 1 mM) and stopped after 15 min with 50 *μ*L malachite green reagent. The activity of either enzyme at the surface of intact Human Umbilical Vein Endothelial Cells (HUVEC, passage 2) or NTPDase1 transfected cells was measured in 24 well plates with the buffers indicated above supplemented with 125 mM NaCl. The reaction was initiated as above and stopped by transferring a 200 *μ*L aliquot of the reaction mixture to a tube containing 50 *μ*L malachite green reagent. The liberated inorganic phosphate (P_i_) was measured at 630 nm according to Baykov et al. [45]. The increase of phosphate concentration due to enzyme activity was calculated by subtracting the phosphate concentration of the control reaction mixture, where the substrate was added after the malachite green reagent, from that of the respective reaction mixture. All experiments were performed in triplicate. One unit of enzymatic activity corresponded to the release of 1 *μ*mol Pi/min*·*mg of protein or 1 *μ*mol Pi/min/well at 37°C for protein extracts and intact cells, respectively. The lysates from nontransfected COS-7 cells exhibited less than 5% of the ATP or ADP hydrolysis generated by lysates from COS-7 cells transfected with either NTPDases' expressing plasmid and as such the activity of the contaminating nucleotidases was considered negligible.

#### 2.4.2. NPPs (EC 3.1.4.1)

Activity assays of human NPP1 and human NPP3 were carried out with paranitrophenyl thymidine 5′-monophosphate (pNP-TMP) as the substrate [42]. pNP-TMP hydrolysis was carried out at 37°C in 0.2 mL of the following incubation mixture: in mM, 1 CaCl_2_, 130 NaCl, 5 KCl, and 50 Tris, pH 8.5, with or without 100 *μ*M ticlopidine. Recombinant human NPP1 or human NPP3 cell lysates were added to the incubation mixture and were preincubated at 37°C for 3 min. The reaction was initiated by the addition of the substrate pNP-TMP (100 *μ*M), with or without 100 *μ*M ticlopidine. The production of* p*-nitrophenol in the reaction medium was measured at 310 nm, 15 min after the initiation of the reaction. The protein extracts from nontransfected COS-7 cells exhibited less than 5% of the pNP-TMP hydrolysis obtained with lysates from either NPP1 or NPP3 transfected cells, and as such was considered negligible.

#### 2.4.3. Enzyme Histochemistry Assays

For histochemical studies, 5 *μ*m sections of freshly dissected tissues were embedded in OCT freezing medium and snap-frozen in isopentane in dry ice and stored at −80°C until use. Sections of 6 *μ*m were obtained and fixed in 10% phosphate-buffered formalin mixed with cold acetone as before [46]. Localization of ectonucleotidase activities was determined using the Wachstein/Meisel lead phosphate method [47]. Fixed slices were preincubated for 30 min at RT in 50 mM Tris-maleate buffer, pH 7.4, containing 2 mM CaCl_2_, 250 mM sucrose, and 2.5 mM levamisole as an inhibitor of alkaline phosphatases. Enzymatic reaction was performed for 1 h at 37°C in the same buffer supplemented with 5 mM MnCl_2_ to inhibit intracellular staining [48], 2 mM Pb(NO_3_)_2_, 3% Dextran T-250 and in the presence of 200 *μ*M ATP with or without 100 *μ*M ticlopidine. For the control experiment, substrate was either omitted or added in the absence of divalent cations, which are essential for NTPDases' activity. The reaction was revealed by incubation with 1% (NH_4_)_2_S v/v for exactly 1 min. Samples were counterstained with aqueous haematoxylin, mounted with Mowiol mounting medium, and visualized and photographed with a BX51 Olympus microscope.

### 2.5. Statistic

Statistical analysis was done with the two-way ANOVA test. *P* values below 0.05 were considered statistically significant.

## 3. Results

### 3.1. Influence of Ticlopidine on NTPDase Activity

**Figure 1:**
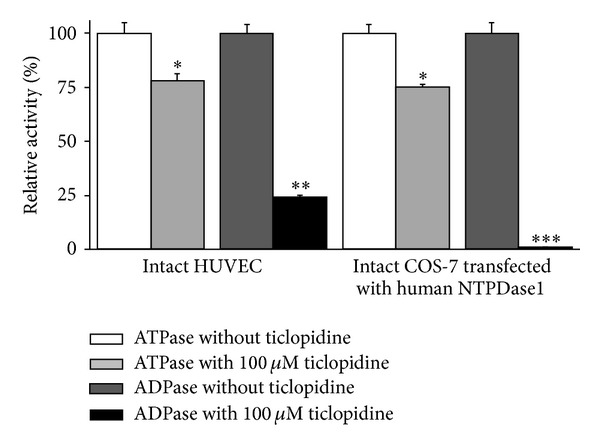
Influence of ticlopidine on intact HUVEC and COS-7 cells transfected with an expression vector encoding human NTPDase1. The substrate (ATP or ADP) was added together with ticlopidine, both at 100 *μ*M. Relative activities are expressed as the mean ± SD of 3 independent experiments with confluent cells (HUVEC from three different donors at passage 2), each performed in triplicate; mean cell number in one well was in the order of 250,000. The activity (without ticlopidine, which was set at 100%) with the substrate ATP corresponded to 2.5 ± 0.12 and 16.7 ± 0.8 nmol P_i_·min^−1^ *·*well^−1^ for HUVEC and transfected COS-7 cells, respectively, and with ADP to 3.5 ± 0.17 and 9.1 ± 0.45 nmol P_i_·min^−1^ *·*well^−1^ for HUVEC and transfected COS-7, respectively. Data are presented as the mean ± SD of 3 experiments carried out in triplicate. *P = 0.04; **P = 0.0003; ***P = 0.0001.

**Figure 2:**
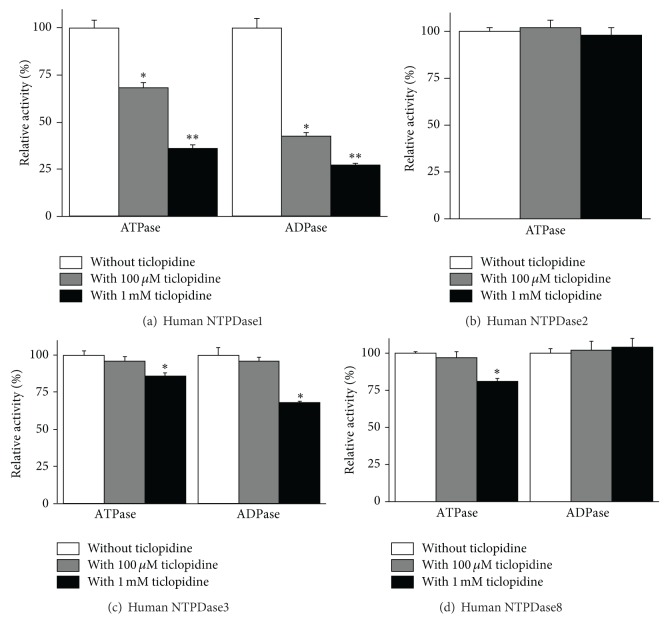
Influence of ticlopidine on recombinant human NTPDase activities. Enzymatic assays were carried out with lysates from COS-7 cells transfected with an expression vector encoding the indicated enzyme. The substrate (ATP or ADP at the concentration of 100 *μ*M) was added alone or together with ticlopidine at the concentration of either 100 *μ*M or 1 mM. The 100% activity in the absence of ticlopidine corresponded to the following: for human NTPDase1 to 670 ± 29 and 550 ± 21 nmol P_i_·min^−1^ *·*mgprotein^−1^ for ATP and ADP as substrates, respectively (a), for human NTPDase2 to 1023 ± 58 nmol P_i_· min^−1^·mgprotein^−1^ for ATP as substrate (b), for human NTPDase3 to 256 ± 37 and 103 ± 10 nmol P_i_·min^−1^·mgprotein^−1^ for ATP and ADP as substrates, respectively (c), and for human NTPDase8 to 148 ± 16 and 33 ± 6 nmol P_i_·min^−1^·mgprotein^−1^ for ATP and ADP as substrates, respectively (d). Data are presented as the mean ± SD of 3 experiments carried out in triplicate. *P = 0.018; **P = 0.0002.

We previously observed that ticlopidine inhibited recombinant human NTPDase1 [46]. In this work we verified if this compound can be used as a specific inhibitor of the enzyme. We first confirmed that ticlopidine was an inhibitor of NTPDase1 from different sources. The ADPase activity of NTPDase1 expressing cells, namely, intact COS-7 cells transfected with human NTPDase1 or HUVEC, was strongly inhibited by 100 *μ*M ticlopidine, 99 and 75% inhibition, respectively (Figure 1). ATPase activity was inhibited by about 25% in both cell types (Figure 1). The same ticlopidine concentration inhibited the ADPase activity of lysates from COS-7 cell transfected with an NTPDase1 expression vector by about 58% (Figure 2(a)), while the ATPase activity was decreased more modestly than the ADPase activity by about 32%, showing a similar tendency than what was observed for intact cells (Figures 1 and 2(a)). One mM ticlopidine further increased the inhibition of ADPase up to 73% and that of ATPase up to 64% (Figure 2(a)). At 100 *μ*M ticlopidine did not impair ATPase or ADPase activities of lysates from COS-7 cells transfected with NTPDase2, NTPDase-3, or NTPDase-8 (Figures 2(b)–2(d)). At 1 mM concentration, ticlopidine inhibited only weakly the ADPase activity (32%) of NTPDase3 as well as its ATPase activity (14%, Figure 2(c)) and the ATPase activity (19%) of NTPDase8 (Figure 2(d)).

### 3.2. Influence of Ticlopidine on Murine NTPDase1 Activity

**Figure 3:**
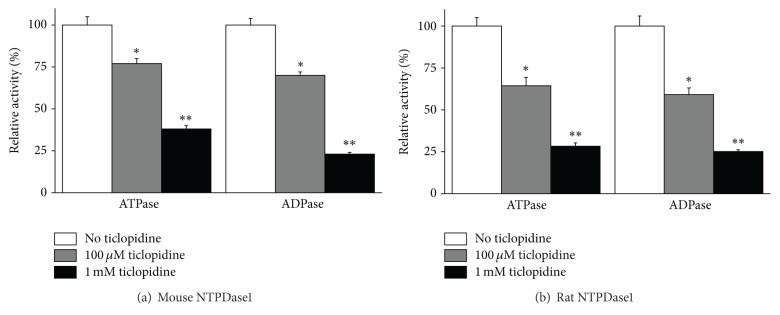
Comparative effect of ticlopidine on recombinant murine NTPDase1. ATPase and ADPase activity of mouse (a) or rat (b) NTPDase1 without and with either 100 *μ*M or 1 mM ticlopidine are presented. The 100% activity with ATP as the substrate in the absence of ticlopidine was 2781 ± 136 and 1502 ± 66 nmol P_i_·min^−1^·mgprotein^−1^ for mouse and rat NTPDase1, respectively, and with ADP 2219 ± 105 and 1103 ± 56 nmol P_i_·min^−1^·mgprotein^−1^ for mouse and rat NTPDase1, respectively. Data are presented as the mean ± SD of 3 experiments carried out in triplicate. *P = 0.0049; **P = 0.0007.

We next investigated whether ticlopidine could also inhibit NTPDase1 from other species. At 100 *μ*M, ticlopidine inhibited the ATPase activity of both, mouse and rat NTPDase1, 23 and 36%, respectively (Figure 3), and ADPase activity by about 30 and 41%, respectively. The inhibition level was similar for all tested species at 1 mM ticlopidine, about 60–70% of ATPase and ~75% of ADPase activity (Figures 2(a) and 3).

### 3.3. Influence of Ticlopidine on Other Ectonucleotidases

**Figure 4:**
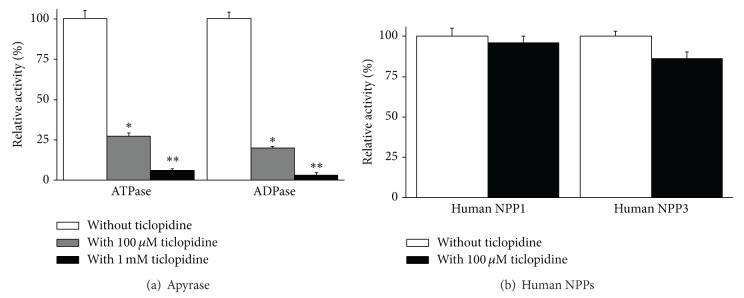
Influence of ticlopidine (100 *μ*M or 1 mM) on the ATPase and ADPase activity of apyrase (a) and (100 *μ*M) on human NPP1 and human NPP3 (b). The activity of apyrase without ticlopidine was 159 ± 7 and 87 ± 4 *μ*moles Pi/min*·*mg protein for ATP and ADP, respectively. Data are presented as the mean ± SD of 3 experiments carried out in triplicate. The 100% activities without ticlopidine with pNP-TMP as the substrate were 71 ± 3 and 17 ± 1 nmol* p*-nitrophenol/min*·*mg protein for NPP1 and NPP3, respectively. *P = 0.0045; **P = 0.0002.

In our previous work we observed that 60 *μ*M ticlopidine, the calculated concentration of the compound after its administration to human patient [46], slightly inhibited rat ecto-5′-nucleotidase but not human ecto-5′-nucleotidase. Here we tested the effect of ticlopidine on other ectonucleotidases, including NTPDase from plant that is commercially available and widely used, namely, potato apyrase. The ADPase activity of this plant NTPDase1 was also slightly more affected than its ATPase activity; the inhibition of ADPase activity by ticlopidine was 80 and 98% at 100 *μ*M and 1 mM ticlopidine, respectively, and its ATPase activity, 75 and 95% for 100 *μ*M and 1 mM ticlopidine, respectively (Figure 4(a)).

There are also 2 other ectonucleotidases, NPPs, that efficiently hydrolyse ATP and ADP: NPP1 and NPP3. For these enzymes we used the synthetic substrate pNP-TMP in our assay. Ticlopidine (100 *μ*M) did not affect the activity of either enzyme (Figure 4(b)). As the NPP activity was tested at a pH (slightly alkaline) that decreases the solubility of ticlopidine, we did not test higher concentrations of ticlopidine.

### 3.4. Ticlopidine Inhibits NTPDase1 Activity in Human Tissues

**Figure 5:**
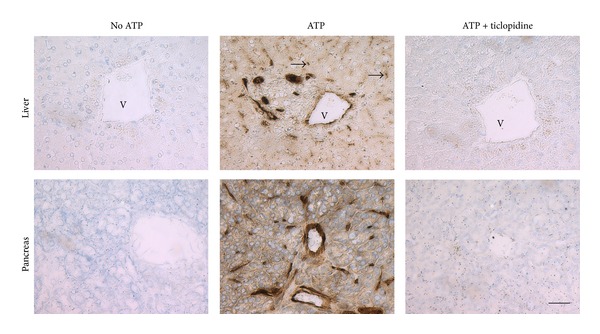
Inhibition of NTPDase1 ATPase activity in human tissues by ticlopidine. Enzyme histochemistry was performed on serial sections with the substrate ATP at a final concentration of 200 *μ*M in the presence or absence of 100 *μ*M ticlopidine. In both tissues (liver and pancreas) NTPDase1 ATPase activity is located in endothelial cells of all blood vessels including capillaries and sinusoids as well as in smooth muscle cells of arteries and in resident macrophages (Küpffer cells in the liver). In addition, in the pancreas NTPDase1 is also expressed at the luminal surface of acinar cells and in zymogen granules. The ATPase activity is seen as a brown precipitate and is completely absent in presence of ticlopidine. Nuclei were counterstained with haematoxylin. Scale bar = 50 *μ*m. V = vein; ∗ = Langerhans islet; arrows = Küpffer cells.

We then tested whether ticlopidine can also inhibit NTPDase1* in situ*. Enzyme histochemistry assays were performed with liver and pancreas tissue sections where NTPDase1 is highly expressed in blood vessels (arteries, veins, capillaries, and sinusoids) as well as in the exocrine cells of the pancreas. Here we have used 200 *μ*M of ATP and 100 *μ*M ticlopidine. Under these conditions ticlopidine abolished the ATPase activity of NTPDase1 (Figure 5). The inhibition observed was even more potent than in assays with cell lysates, similar to what we measured with the experiments with intact cells (Figures 1, 2(a), and 5).

## 4. Discussion

By regulating extracellular nucleotide levels, NTPDase1 affects haemostasis [12, 13, 37], leukocyte migration [24, 25], immune responses [6, 49], angiogenesis, vascular permeability [17, 50], and vasoconstriction [8, 15]. Therefore the identification of selective NTPDase1 inhibitors would be valuable tools to study the function and pathological consequence of dysregulation of NTPDase1 activity. Additionally, changes in ATP and ADP levels, potent ecto-5′-nucleotidase inhibitors, change the level of adenosine and modulate the physiological responses of P1 receptor activation for which adenosine is the agonist [33, 51].

Some inhibitors of NTPDase1 have been described and characterized. Unfortunately most of them are not specific as they also inhibit other ectonucleotidases or affect purinoceptor activity. *N* ^6^,*N* ^6^-diethyl-D-*β*-*γ*-dibromomethylene-ATP, also named ARL 67156, was found to be a weak and nonselective NPP1, NTPDase1, and NTPDase3 inhibitor [52, 53]. Polyoxometalate (POM-1) inhibits NTPDase1 but its action is limited by off-target actions on synaptic transmission [53, 54]. 1-amino-2-sulfo-4-(2-naphthylamino) anthraquinone was shown as a potent inhibitor of NTPDase1 but it inhibited at a similar level NTPDase3 [55]; suramin and sulfonate dyes such as reactive blue and pyridoxal phosphate-6-azophenyl-2′,4′-disulfonic acid (PPADS) are also nonspecific inhibitors of NTPDase1 activity [31, 56–59]. Recently we have synthesized and characterized potent and selective inhibitors of NTPDase1 that are analogues of adenine nucleotides, namely, 8-BuS-ADP and 8-BuS-AMP [46]. Here we report that ticlopidine is also a potent and selective inhibitor of NTPDase1 and as such can be used as a tool to study this ectonucleotidase function and pathophysiological consequences of abnormal activity. Ticlopidine is routinely administered to patients as a part of an antithrombotic therapy [60], but before it is activated by the liver it does not activate nor antagonize P2 receptors [61]. In a previous work we showed that thienopyridines, in their respective prodrug forms, prevent NTPDase1 antiplatelet activity, due to the inhibition of its ADPase activity [33]. 100 *μ*M ticlopidine inhibited the hydrolysis of ADP by about 80% [33]. Kinetic assays of the recombinant NTPDase1 showed a mixed type inhibition by ticlopidine with a *K*_iapp_ of 14 [33]. Here we further showed that ticlopidine can be used as a specific inhibitor of NTPDase1 from various species, human, mouse, and rat (Figures 2(a) and 3). At 100 *μ*M concentration, ticlopidine inhibited the murine forms of NTPDase1 less efficiently than human NTPDase1, but at 1 mM we observed the same level of inhibition for all tested species (Figures 2(a) and 3). The most important value of this molecule is its selectivity in its prodrug form. Indeed at 100 *μ*M, ticlopidine did not affect the activity of the other major ectonucleotidases, namely, NTPDase2, NTPDase3, NTPDase8, NPP1, and NPP3 (Figures 2(b)–2(d) and 4), whereas at 1 mM, ticlopidine weakly impeded the activities of NTPDase3 and the ATPase of NTPDase8 (10–20% inhibition, Figures 2(c) and 2(d)). In our previous study, we saw that ticlopidine had no effects on the human ecto-5′-nucleotidase activity and decreased the activity of rat ecto-5′-nucleotidase by about 25% at the concentration of 300 *μ*M [33].

An interesting aspect in the actual study was the observation that the inhibition of NTPDase1 was more striking with a near complete inhibition when the enzyme was tested in its intact natural form, at the surface of cells such as in HUVEC or in tissues compared to the recombinant enzyme from a cell lysate (Figures 1 and 5). This characteristic, which needs to be further investigated, makes ticlopidine a good candidate for inhibition assays with cells that express NTPDase1.

In summary, we identified ticlopidine as a new specific inhibitor of NTPDase1 that is specifically efficient with cell expressed NTPDase1.
